# Benzyl *N*-(2-hy­droxy-1-{*N*′-[(1*E*)-2-hy­droxy­benzyl­idene]hydrazinecarbon­yl}eth­yl)carbamate

**DOI:** 10.1107/S1600536811025128

**Published:** 2011-06-30

**Authors:** Edward R. T. Tiekink, Marcus V. N. de Souza, Alessandra C. Pinheiro, Solange M. S. V. Wardell, James L. Wardell

**Affiliations:** a Department of Chemistry, University of Malaya, 50603 Kuala Lumpur, Malaysia; b Fundação Oswaldo Cruz, Instituto de Tecnologia, em Fármacos – Farmanguinhos, R. Sizenando Nabuco, 100, Manguinhos, 21041-250 Rio de Janeiro, RJ, Brazil; c CHEMSOL, 1 Harcourt Road, Aberdeen AB15 5NY, Scotland; d Centro de Desenvolvimento Tecnológico em Saúde (CDTS), Fundação Oswaldo Cruz (FIOCRUZ), Casa Amarela, Campus de Manguinhos, Av. Brasil 4365, 21040-900 Rio de Janeiro, RJ, Brazil

## Abstract

The mol­ecule of the title compound, C_18_H_19_N_3_O_5_, adopts a curved arrangement with the terminal benzene rings lying to the same side. The hydroxyl­benzene ring is close to coplanar with the adjacent hydrazine residue [dihedral angle = 11.14 (12)°], an observation which correlates with the presence of an intra­molecular O—H⋯N hydrogen bond. The benzyl ring forms a dihedral angle of 50.84 (13)° with the adjacent carbamate group. A twist in the mol­ecule, at the chiral C atom, is reflected in the dihedral angle of 80.21 (12)° formed between the amide residues. In the crystal, two-dimensional arrays in the *ac* plane are mediated by O—H⋯O and N—H⋯O hydrogen bonds.

## Related literature

For background to the use of l-serine derivatives in anti-tumour therapy, see: Jiao *et al.* (2009 ▶); Yakura *et al.* (2007 ▶). For background to *N*-acyl­hydrazone derivatives from l-serine for anti-tumour testing, see: Pinheiro *et al.* (2010 ▶, 2011*a* ▶,*b* ▶); de Souza *et al.* (2010 ▶, 2011 ▶); Howie *et al.* (2011 ▶).
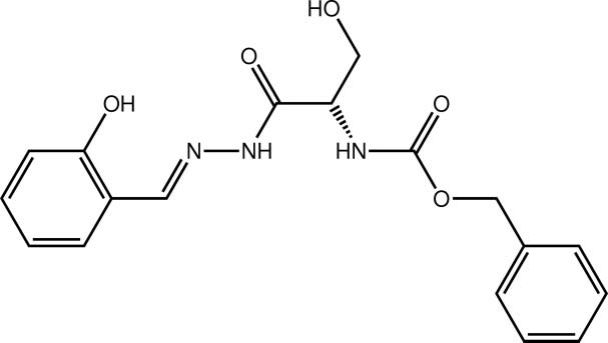


## Experimental

### 

#### Crystal data

C_18_H_19_N_3_O_5_
*M* *_r_* = 357.36
Monoclinic, 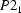

*a* = 5.0338 (5) Å
*b* = 31.357 (3) Å
*c* = 5.5882 (6) Å
β = 97.890 (3)°
*V* = 873.72 (16) Å^3^
*Z* = 2
Mo *K*α radiation
μ = 0.10 mm^−1^
*T* = 120 K
0.25 × 0.05 × 0.02 mm

#### Data collection

Bruker–Nonius Roper CCD camera on κ-goniostat diffractometer
Absorption correction: multi-scan (*SADABS*; Sheldrick, 2007 ▶) *T*_min_ = 0.654, *T*_max_ = 0.746
8786 measured reflections
2034 independent reflections
1485 reflections with *I* > 2σ(*I*)
*R*_int_ = 0.066

#### Refinement

*R*[*F* ^2^ > 2σ(*F* ^2^)] = 0.053
*wR*(*F* ^2^) = 0.098
*S* = 1.06
2034 reflections
247 parameters
1 restraint
H atoms treated by a mixture of independent and constrained refinement
Δρ_max_ = 0.17 e Å^−3^
Δρ_min_ = −0.20 e Å^−3^

### 

Data collection: *COLLECT* (Hooft, 1998 ▶); cell refinement: *DENZO* (Otwinowski & Minor, 1997 ▶) and *COLLECT*; data reduction: *DENZO* and *COLLECT*; program(s) used to solve structure: *SHELXS97* (Sheldrick, 2008 ▶); program(s) used to refine structure: *SHELXL97* (Sheldrick, 2008 ▶); molecular graphics: *ORTEP-3* (Farrugia, 1997 ▶) and *DIAMOND* (Brandenburg, 2006 ▶); software used to prepare material for publication: *publCIF* (Westrip, 2010 ▶).

## Supplementary Material

Crystal structure: contains datablock(s) global, I. DOI: 10.1107/S1600536811025128/hb5935sup1.cif

Structure factors: contains datablock(s) I. DOI: 10.1107/S1600536811025128/hb5935Isup2.hkl

Additional supplementary materials:  crystallographic information; 3D view; checkCIF report

## Figures and Tables

**Table 1:** Hydrogen-bond geometry (Å, °)

*D*—H⋯*A*	*D*—H	H⋯*A*	*D*⋯*A*	*D*—H⋯*A*
O1—H1o⋯N1	0.90 (5)	1.88 (5)	2.648 (4)	143 (4)
O3—H3o⋯O2^i^	0.84 (5)	1.79 (5)	2.616 (4)	167 (5)
N2—H2n⋯O3^ii^	0.90 (4)	1.87 (4)	2.758 (4)	168 (4)
N3—H3n⋯O4^iii^	0.95 (4)	1.97 (4)	2.897 (4)	165 (3)

Symmetry codes: (i) 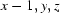; (ii) 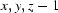; (iii) 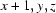.

**Fig. 1.**
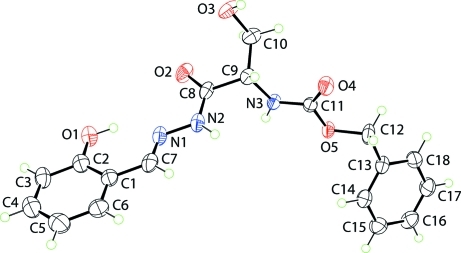
The molecular structure of (I) showing displacement ellipsoids at the 50% probability level.

**Fig. 2.**
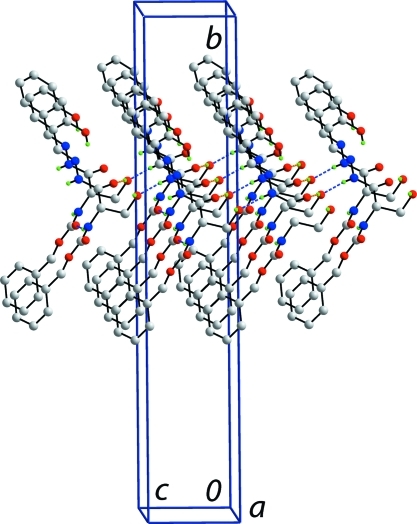
A view of the supramolecular array in the ac plane in (I) with the O—H···O and N—H···O hydrogen bonding shown as orange and blue dashed lines, respectively. Hydrogen atoms not participating in the hydrogen bonding scheme are omitted for reasons of clariy.

**Fig. 3.**
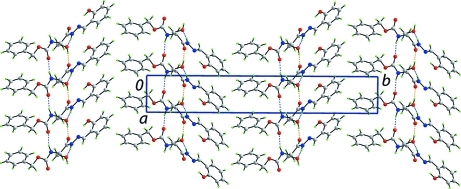
A view in projection down the c axis of the stacking of 2-D supramolecular arrays alng the b axis in (I), and with the O—H···O and N—H···O hydrogen bonding shown as orange and blue dashed lines, respectively.

## supplementary crystallographic information

### Comment

The known anti-tumour activity of *L*-serine derivatives (Jiao *et al.*, 2009; Yakura *et al.*,2007) motivates the development of *N*-acylhydrazone derivatives from *L*-serine for anti-tumour testing (Pinheiro *et al.*, 2010; de Souza *et al.*, 2010; Pinheiro *et al.*, 2011*a*; Pinheiro *et al.*, 2011*b*, de Souza *et al.*, 2011; Howie *et al.*, 2011), and led to the analysis of (I).

The absolute structure of (I) could not be determined experimentally but, the assignment of the *S*-configuration at the C9 atom is based on a starting reagent, *L*-serine. The structure of (I), Fig. 1, adopts a curved conformation with both benzene rings lying to the same side of the molecule. The presence of an intramolecular O—H···N hydrogen bond ensures that the hydroxybenzene group is co-planar with the adjacent hydrazine residue with the dihedral angle between the (O2,N1,N2,C7,C8) and (C1–C6) planes being 11.14 (12) °. By contrast, the benzene ring adjacent to the carbamate residue is twisted as seen in the value of the dihedral angle formed between (O4,O5,N3,C11) and (C13–C18) of 50.84 (13) °. The dihedral angle between the two terminal benzene rings is 75.89 (19) °. The molecule is twisted about the chiral centre with the dihedral angle formed between the two amide residues, *i.e*. N2,C8,O2 and N3,C11,O4, being 80.21 (12) °.

Hydrogen bonds dominate the crystal packing, Table 1. Thus, the secondary hydroxyl group forms a O—H···O hydrogen bond to the hydrazine-carbonyl-O2, and accepts a N—H···.O hydrogen bond from the hydrazine-amine, leading to chains along the *c* axis. The carbamate-amine forms a N—H···O hydrogen bond to the carbamate-carbonyl-O4, leading to chains along the *a* axis. The result is the formation of a two-dimensional array in the *ac* plane, Fig. 2. The layers stack along the *b* axis, Fig. 3.

### Experimental

To a stirred solution of methyl (2*S*)-2-[(benzyloxycarbonyl)amino]-3-hydroxypropanoate (0.3 g, 1.17 mmol), prepared from (2*S*)-2-amino-3-hydroxypropanoate hydrochloride and benzyl chloroformate (21 ml, 0.15 mol), in ethanol (10 ml) was added N_2_H_4_.H_2_O (80%, 5.5 mmol). The reaction mixture was stirred for 24 h at room temperature, rotary evaporated and the residue washed with cold ethanol (3 *x* 10 ml) to give benzyl (1*S*)-2-hydrazino-1-(hydroxymethyl)-2-oxoethylcarbamate in 78% yield, which was used as such for the next stage. To a stirred solution of (*S*)-PhCH_2_OCONHCH(CH_2_OH)CONHNH_2_ (1.0 mmol) in ethanol (10 ml) at room temperature was added 2-hydroxybenzaldehyde (1.05 mmol). The reaction mixture was refluxed for 4 h, rotary evaporated and the residue purified by washing with cold ethanol (3 *x* 10 ml), affording the title compound, *M*.pt. 433 K, yield 89%. The sample for the structure determination was recrystallized from EtOH to yield colourless laths. ^1^H NMR (500 MHz, DMSO-d6) δ (p.p.m.): 11.79 (1*H*, s, NHN), 11.12 (1*H*, s, Ph—OH), 8.46 (1*H*, s, N═ CH, (*E*)-diastereomer), 7.52 (1*H*, dd, J = 7.8 and J = 1.5, H6), 7.48 (1*H*, d, J = 7.8, NHCH), 7.40–7.20 (6*H*, m, Ph and (H4 or H5)), 6.93–6.84 (2*H*, m, H3 and (H4 or H5)), 5.05 (3*H*, m, CH2Ph and OH), 4.13 (1*H*, m, CH), 3.80–3.60 (2*H*, m, CH_2_OH). ^13^C NMR (125 MHz, DMSO-d6) δ (p.p.m.): 171.6, 157.8, 156.4, 141.2, 137.4, 131.9, 131.6, 129.8, 128.8, 128.3, 128.2, 126.7, 119.9, 119.1, 116.6, 65.9, 61.5, 55.0. IR (cm^-1^, KBr): 3312 ν(O—H), 1681 ν(COCH and COO). MS/ESI: [M—H]: 356.3.

### Refinement

The C-bound H atoms were geometrically placed (C–H = 0.95–1.00 Å) and refined as riding with *U*_iso_(H) = 1.2–1.5*U*_eq_(C). The O– and N-bound H atoms were located from a difference map and refined with the distance restraints O–H = 0.84 ± 0.01 and N–H = 0.88±0.01 Å, and with *U*_iso_(H) = *zU*_eq_(carrier atom); *z* = 1.5 for O and *z* = 1.2 for N. In the absence of significant anomalous scattering effects, 1896 Friedel pairs were averaged in the final refinement. However, the absolute configuration was assigned on the basis of the chirality of the *L*-serine starting material.

### Crystal data

**Table:**

C_18_H_19_N_3_O_5_	*F*(000) = 376
*M**_r_* = 357.36	*D*_x_ = 1.358 Mg m^−^^3^
Monoclinic, *P*2_1_	Mo *K*α radiation, λ = 0.71073 Å
Hall symbol: P 2yb	Cell parameters from 11033 reflections
*a* = 5.0338 (5) Å	θ = 2.9–27.5°
*b* = 31.357 (3) Å	µ = 0.10 mm^−^^1^
*c* = 5.5882 (6) Å	*T* = 120 K
β = 97.890 (3)°	Lath, colourless
*V* = 873.72 (16) Å^3^	0.25 × 0.05 × 0.02 mm
*Z* = 2	

### Data collection

**Table:**

Bruker–Nonius Roper CCD camera on κ-goniostat diffractometer	2034 independent reflections
Radiation source: Bruker-Nonius FR591 rotating anode	1485 reflections with *I* > 2σ(*I*)
graphite	*R*_int_ = 0.066
Detector resolution: 9.091 pixels mm^-1^	θ_max_ = 27.5°, θ_min_ = 3.7°
φ & ω scans	*h* = −6→6
Absorption correction: multi-scan (*SADABS*; Sheldrick, 2007)	*k* = −40→40
*T*_min_ = 0.654, *T*_max_ = 0.746	*l* = −7→7
8786 measured reflections	

### Refinement

**Table:**

Refinement on *F*^2^	Secondary atom site location: difference Fourier map
Least-squares matrix: full	Hydrogen site location: inferred from neighbouring sites
*R*[*F*^2^ > 2σ(*F*^2^)] = 0.053	H atoms treated by a mixture of independent and constrained refinement
*wR*(*F*^2^) = 0.098	*w* = 1/[σ^2^(*F*_o_^2^) + (0.0303*P*)^2^ + 0.1614*P*] where *P* = (*F*_o_^2^ + 2*F*_c_^2^)/3
*S* = 1.06	(Δ/σ)_max_ < 0.001
2034 reflections	Δρ_max_ = 0.17 e Å^−^^3^
247 parameters	Δρ_min_ = −0.20 e Å^−^^3^
1 restraint	Absolute structure: nd
Primary atom site location: structure-invariant direct methods	

### Special details

**Table:**

Geometry. All s.u.'s (except the s.u. in the dihedral angle between two l.s. planes) are estimated using the full covariance matrix. The cell s.u.'s are taken into account individually in the estimation of s.u.'s in distances, angles and torsion angles; correlations between s.u.'s in cell parameters are only used when they are defined by crystal symmetry. An approximate (isotropic) treatment of cell s.u.'s is used for estimating s.u.'s involving l.s. planes.
Refinement. Refinement of *F*^2^ against ALL reflections. The weighted *R*-factor *wR* and goodness of fit *S* are based on *F*^2^, conventional *R*-factors *R* are based on *F*, with *F* set to zero for negative *F*^2^. The threshold expression of *F*^2^ > 2σ(*F*^2^) is used only for calculating *R*-factors(gt) *etc*. and is not relevant to the choice of reflections for refinement. *R*-factors based on *F*^2^ are statistically about twice as large as those based on *F*, and *R*- factors based on ALL data will be even larger.

### Fractional atomic coordinates and isotropic or equivalent isotropic displacement parameters (Å^2^)

**Table:**

	*x*	*y*	*z*	*U*_iso_*/*U*_eq_	
O1	0.7558 (5)	0.24963 (8)	0.4299 (5)	0.0409 (7)	
H1O	0.629 (9)	0.2299 (15)	0.440 (9)	0.061*	
O2	0.2652 (5)	0.16299 (9)	0.6800 (5)	0.0427 (7)	
O3	−0.2389 (5)	0.15356 (10)	0.8847 (5)	0.0443 (7)	
H3O	−0.401 (10)	0.1591 (16)	0.838 (8)	0.066*	
O4	−0.5493 (5)	0.07490 (8)	0.2844 (5)	0.0404 (7)	
O5	−0.2450 (4)	0.03271 (7)	0.1329 (4)	0.0303 (6)	
N1	0.3164 (6)	0.20615 (9)	0.2693 (6)	0.0343 (7)	
N2	0.1183 (6)	0.17580 (9)	0.2843 (6)	0.0331 (7)	
H2N	−0.011 (8)	0.1717 (13)	0.159 (7)	0.040*	
N3	−0.1010 (6)	0.08812 (9)	0.3608 (5)	0.0274 (7)	
H3N	0.069 (7)	0.0788 (11)	0.327 (6)	0.033*	
C1	0.4747 (7)	0.26400 (11)	0.0505 (7)	0.0353 (9)	
C2	0.6973 (7)	0.27309 (12)	0.2247 (8)	0.0366 (9)	
C3	0.8652 (8)	0.30728 (12)	0.1904 (8)	0.0412 (9)	
H3	1.0135	0.3136	0.3093	0.049*	
C4	0.8165 (9)	0.33195 (13)	−0.0161 (8)	0.0472 (11)	
H4	0.9325	0.3551	−0.0386	0.057*	
C5	0.5994 (9)	0.32322 (14)	−0.1910 (8)	0.0506 (11)	
H5	0.5663	0.3403	−0.3322	0.061*	
C6	0.4311 (8)	0.28924 (13)	−0.1577 (8)	0.0439 (10)	
H6	0.2841	0.2830	−0.2783	0.053*	
C7	0.2850 (7)	0.22982 (12)	0.0788 (8)	0.0366 (9)	
H7	0.1376	0.2250	−0.0437	0.044*	
C8	0.1053 (6)	0.15684 (11)	0.4959 (6)	0.0304 (8)	
C9	−0.1351 (7)	0.12663 (11)	0.4999 (6)	0.0287 (8)	
H9	−0.2995	0.1417	0.4215	0.034*	
C10	−0.1744 (7)	0.11639 (12)	0.7568 (7)	0.0367 (9)	
H10A	−0.3207	0.0953	0.7555	0.044*	
H10B	−0.0082	0.1035	0.8416	0.044*	
C11	−0.3170 (7)	0.06639 (11)	0.2608 (6)	0.0292 (8)	
C12	−0.4669 (8)	0.00648 (12)	0.0233 (8)	0.0430 (10)	
H12A	−0.6019	0.0244	−0.0757	0.052*	
H12B	−0.5545	−0.0076	0.1503	0.052*	
C13	−0.3575 (7)	−0.02651 (11)	−0.1330 (7)	0.0334 (9)	
C14	−0.1860 (8)	−0.01563 (12)	−0.2975 (7)	0.0384 (9)	
H14	−0.1289	0.0131	−0.3075	0.046*	
C15	−0.0973 (9)	−0.04597 (13)	−0.4467 (7)	0.0451 (10)	
H15	0.0198	−0.0380	−0.5587	0.054*	
C16	−0.1781 (8)	−0.08796 (12)	−0.4336 (7)	0.0435 (10)	
H16	−0.1178	−0.1089	−0.5368	0.052*	
C17	−0.3479 (8)	−0.09931 (13)	−0.2687 (7)	0.0445 (10)	
H17	−0.4039	−0.1281	−0.2588	0.053*	
C18	−0.4360 (8)	−0.06899 (12)	−0.1191 (7)	0.0414 (10)	
H18	−0.5510	−0.0771	−0.0057	0.050*	

### Atomic displacement parameters (Å^2^)

**Table:**

	*U*^11^	*U*^22^	*U*^33^	*U*^12^	*U*^13^	*U*^23^
O1	0.0346 (15)	0.0338 (15)	0.0538 (17)	−0.0029 (12)	0.0040 (13)	0.0000 (14)
O2	0.0275 (13)	0.0537 (18)	0.0434 (15)	0.0004 (12)	−0.0074 (12)	−0.0141 (13)
O3	0.0277 (13)	0.0647 (19)	0.0378 (15)	0.0139 (14)	−0.0050 (11)	−0.0248 (14)
O4	0.0199 (12)	0.0454 (16)	0.0569 (17)	−0.0006 (11)	0.0091 (12)	−0.0179 (14)
O5	0.0221 (12)	0.0292 (14)	0.0398 (14)	−0.0031 (10)	0.0048 (11)	−0.0104 (11)
N1	0.0247 (15)	0.0287 (16)	0.049 (2)	−0.0013 (13)	0.0051 (14)	−0.0106 (16)
N2	0.0261 (16)	0.0280 (16)	0.044 (2)	−0.0023 (13)	0.0009 (14)	−0.0070 (14)
N3	0.0178 (14)	0.0290 (16)	0.0353 (16)	0.0022 (12)	0.0032 (13)	−0.0097 (13)
C1	0.035 (2)	0.030 (2)	0.044 (2)	0.0020 (16)	0.0139 (18)	−0.0081 (17)
C2	0.033 (2)	0.0279 (19)	0.051 (2)	0.0053 (16)	0.0136 (18)	−0.0052 (19)
C3	0.034 (2)	0.030 (2)	0.060 (3)	−0.0013 (18)	0.0095 (19)	−0.005 (2)
C4	0.044 (2)	0.033 (2)	0.069 (3)	0.0024 (18)	0.023 (2)	−0.005 (2)
C5	0.056 (3)	0.042 (2)	0.057 (3)	0.007 (2)	0.021 (2)	0.001 (2)
C6	0.047 (2)	0.042 (2)	0.045 (2)	0.0035 (19)	0.014 (2)	−0.0073 (19)
C7	0.032 (2)	0.030 (2)	0.049 (2)	0.0038 (15)	0.0080 (18)	−0.0136 (19)
C8	0.0210 (17)	0.0295 (19)	0.039 (2)	0.0042 (15)	−0.0030 (15)	−0.0110 (17)
C9	0.0230 (17)	0.0299 (19)	0.0321 (19)	0.0057 (15)	−0.0003 (15)	−0.0035 (15)
C10	0.0291 (19)	0.046 (2)	0.034 (2)	0.0114 (17)	0.0007 (16)	−0.0069 (18)
C11	0.0248 (19)	0.0317 (19)	0.0318 (19)	−0.0024 (15)	0.0066 (15)	−0.0072 (16)
C12	0.028 (2)	0.040 (2)	0.061 (3)	−0.0096 (17)	0.006 (2)	−0.022 (2)
C13	0.0280 (19)	0.029 (2)	0.042 (2)	−0.0010 (15)	0.0006 (17)	−0.0080 (17)
C14	0.042 (2)	0.030 (2)	0.042 (2)	−0.0026 (17)	0.0029 (18)	−0.0054 (17)
C15	0.059 (3)	0.037 (2)	0.041 (2)	−0.003 (2)	0.013 (2)	−0.0047 (19)
C16	0.057 (3)	0.031 (2)	0.043 (2)	0.0049 (18)	0.006 (2)	−0.0081 (19)
C17	0.052 (2)	0.029 (2)	0.053 (3)	−0.0063 (19)	0.010 (2)	−0.0085 (19)
C18	0.042 (2)	0.039 (2)	0.045 (2)	−0.0071 (18)	0.0127 (19)	−0.0061 (19)

### Geometric parameters (Å, °)

**Table:**

O1—C2	1.360 (5)	C5—C6	1.389 (6)
O1—H1O	0.89 (5)	C5—H5	0.9500
O2—C8	1.231 (4)	C6—H6	0.9500
O3—C10	1.428 (4)	C7—H7	0.9500
O3—H3O	0.84 (5)	C8—C9	1.540 (5)
O4—C11	1.224 (4)	C9—C10	1.510 (5)
O5—C11	1.352 (4)	C9—H9	1.0000
O5—C12	1.453 (4)	C10—H10A	0.9900
N1—C7	1.290 (5)	C10—H10B	0.9900
N1—N2	1.389 (4)	C12—C13	1.506 (5)
N2—C8	1.333 (5)	C12—H12A	0.9900
N2—H2N	0.90 (4)	C12—H12B	0.9900
N3—C11	1.339 (4)	C13—C14	1.387 (6)
N3—C9	1.459 (4)	C13—C18	1.395 (5)
N3—H3N	0.95 (4)	C14—C15	1.379 (5)
C1—C6	1.400 (5)	C14—H14	0.9500
C1—C2	1.409 (5)	C15—C16	1.383 (6)
C1—C7	1.458 (5)	C15—H15	0.9500
C2—C3	1.394 (5)	C16—C17	1.387 (6)
C3—C4	1.383 (6)	C16—H16	0.9500
C3—H3	0.9500	C17—C18	1.379 (5)
C4—C5	1.390 (6)	C17—H17	0.9500
C4—H4	0.9500	C18—H18	0.9500
			
C2—O1—H1O	111 (3)	N3—C9—H9	108.0
C10—O3—H3O	107 (3)	C10—C9—H9	108.0
C11—O5—C12	114.7 (2)	C8—C9—H9	108.0
C7—N1—N2	115.9 (3)	O3—C10—C9	111.8 (3)
C8—N2—N1	118.8 (3)	O3—C10—H10A	109.3
C8—N2—H2N	121 (3)	C9—C10—H10A	109.3
N1—N2—H2N	120 (3)	O3—C10—H10B	109.3
C11—N3—C9	119.7 (3)	C9—C10—H10B	109.3
C11—N3—H3N	118 (2)	H10A—C10—H10B	107.9
C9—N3—H3N	122 (2)	O4—C11—N3	125.3 (3)
C6—C1—C2	118.6 (3)	O4—C11—O5	123.8 (3)
C6—C1—C7	118.4 (4)	N3—C11—O5	110.9 (3)
C2—C1—C7	123.0 (4)	O5—C12—C13	108.2 (3)
O1—C2—C3	117.8 (4)	O5—C12—H12A	110.1
O1—C2—C1	122.2 (3)	C13—C12—H12A	110.1
C3—C2—C1	120.0 (4)	O5—C12—H12B	110.1
C4—C3—C2	120.2 (4)	C13—C12—H12B	110.1
C4—C3—H3	119.9	H12A—C12—H12B	108.4
C2—C3—H3	119.9	C14—C13—C18	118.6 (3)
C3—C4—C5	120.6 (4)	C14—C13—C12	121.7 (3)
C3—C4—H4	119.7	C18—C13—C12	119.7 (4)
C5—C4—H4	119.7	C15—C14—C13	120.9 (4)
C4—C5—C6	119.5 (4)	C15—C14—H14	119.5
C4—C5—H5	120.3	C13—C14—H14	119.5
C6—C5—H5	120.3	C14—C15—C16	120.2 (4)
C5—C6—C1	121.1 (4)	C14—C15—H15	119.9
C5—C6—H6	119.5	C16—C15—H15	119.9
C1—C6—H6	119.5	C15—C16—C17	119.5 (4)
N1—C7—C1	120.3 (3)	C15—C16—H16	120.3
N1—C7—H7	119.8	C17—C16—H16	120.3
C1—C7—H7	119.8	C18—C17—C16	120.3 (4)
O2—C8—N2	124.4 (3)	C18—C17—H17	119.8
O2—C8—C9	120.6 (3)	C16—C17—H17	119.8
N2—C8—C9	114.9 (3)	C17—C18—C13	120.5 (4)
N3—C9—C10	111.8 (3)	C17—C18—H18	119.7
N3—C9—C8	110.6 (3)	C13—C18—H18	119.7
C10—C9—C8	110.5 (3)		
			
C7—N1—N2—C8	−165.5 (3)	N2—C8—C9—N3	71.1 (4)
C6—C1—C2—O1	−178.9 (3)	O2—C8—C9—C10	13.6 (4)
C7—C1—C2—O1	2.0 (5)	N2—C8—C9—C10	−164.7 (3)
C6—C1—C2—C3	1.5 (5)	N3—C9—C10—O3	−173.2 (3)
C7—C1—C2—C3	−177.6 (3)	C8—C9—C10—O3	63.2 (3)
O1—C2—C3—C4	179.4 (3)	C9—N3—C11—O4	−3.6 (5)
C1—C2—C3—C4	−1.0 (5)	C9—N3—C11—O5	177.9 (3)
C2—C3—C4—C5	0.4 (6)	C12—O5—C11—O4	−0.2 (5)
C3—C4—C5—C6	−0.3 (6)	C12—O5—C11—N3	178.3 (3)
C4—C5—C6—C1	0.8 (6)	C11—O5—C12—C13	174.0 (3)
C2—C1—C6—C5	−1.4 (5)	O5—C12—C13—C14	−48.4 (5)
C7—C1—C6—C5	177.7 (3)	O5—C12—C13—C18	133.7 (4)
N2—N1—C7—C1	177.1 (3)	C18—C13—C14—C15	0.9 (6)
C6—C1—C7—N1	−179.2 (3)	C12—C13—C14—C15	−177.1 (4)
C2—C1—C7—N1	−0.2 (5)	C13—C14—C15—C16	−0.2 (6)
N1—N2—C8—O2	−3.2 (5)	C14—C15—C16—C17	−0.3 (6)
N1—N2—C8—C9	174.9 (3)	C15—C16—C17—C18	0.1 (7)
C11—N3—C9—C10	82.9 (4)	C16—C17—C18—C13	0.6 (6)
C11—N3—C9—C8	−153.6 (3)	C14—C13—C18—C17	−1.0 (6)
O2—C8—C9—N3	−110.7 (4)	C12—C13—C18—C17	177.0 (4)

### Hydrogen-bond geometry (Å, °)

**Table:**

*D*—H···*A*	*D*—H	H···*A*	*D*···*A*	*D*—H···*A*
O1—H1o···N1	0.90 (5)	1.88 (5)	2.648 (4)	143 (4)
O3—H3o···O2^i^	0.84 (5)	1.79 (5)	2.616 (4)	167 (5)
N2—H2n···O3^ii^	0.90 (4)	1.87 (4)	2.758 (4)	168 (4)
N3—H3n···O4^iii^	0.95 (4)	1.97 (4)	2.897 (4)	165 (3)

Symmetry codes: (i) *x*−1, *y*, *z*; (ii) *x*, *y*, *z*−1; (iii) *x*+1, *y*, *z*.
